# Genome-Wide Investigation of N6-Methyladenosine Regulatory Genes and Their Roles in Tea (*Camellia sinensis*) Leaves During Withering Process

**DOI:** 10.3389/fpls.2021.702303

**Published:** 2021-06-15

**Authors:** Chen Zhu, Shuting Zhang, Chengzhe Zhou, Siyi Xie, Guangwu Chen, Caiyun Tian, Kai Xu, Yuling Lin, Zhongxiong Lai, Yuqiong Guo

**Affiliations:** ^1^College of Horticulture, Fujian Agriculture and Forestry University, Fuzhou, China; ^2^Institute of Horticultural Biotechnology, Fujian Agriculture and Forestry University, Fuzhou, China; ^3^Key Laboratory of Tea Science in Universities of Fujian Province, Fujian Agriculture and Forestry University, Fuzhou, China; ^4^Tea Industry Research Institute, Fujian Agriculture and Forestry University, Fuzhou, China

**Keywords:** RNA methylation, *Camellia sinensis*, abiotic stress, withering process, flavonoid

## Abstract

N6-methyladenosine (m6A), one of the internal modifications of RNA molecules, can directly influence RNA abundance and function without altering the nucleotide sequence, and plays a pivotal role in response to diverse environmental stresses. The precise m6A regulatory mechanism comprises three types of components, namely, m6A writers, erasers, and readers. To date, the research focusing on m6A regulatory genes in plant kingdom is still in its infancy. Here, a total of 34 m6A regulatory genes were identified from the chromosome-scale genome of tea plants. The expansion of m6A regulatory genes was driven mainly by whole-genome duplication (WGD) and segmental duplication, and the duplicated gene pairs evolved through purifying selection. Gene structure analysis revealed that the sequence variation contributed to the functional diversification of m6A regulatory genes. Expression pattern analysis showed that most m6A regulatory genes were differentially expressed under environmental stresses and tea-withering stage. These observations indicated that m6A regulatory genes play essential roles in response to environmental stresses and tea-withering stage. We also found that RNA methylation and DNA methylation formed a negative feedback by interacting with each other’s methylation regulatory genes. This study provided a foundation for understanding the m6A-mediated regulatory mechanism in tea plants under environmental stresses and tea-withering stage.

## Introduction

Methylation, one of the representative epigenetic modifications, can directly influence gene expression and function without altering the gene sequence. At the molecular level, DNA methylation is a pervasive modification that plays a broad role in myriad biological processes. Recently, extensive studies have shown that methylation occurs not only on DNAs but also on RNAs (Fu et al., 2014; Yang et al., 2018). RNA methylation is one of the internal modifications of RNA molecules. Among diverse methylation types found on RNAs, N6-methyladenosine (m6A) is the best-characterized and most predominant form of eukaryotic RNA methylation, accounting for up to 80% of RNA methylation in animals and plants. Similar to DNA methylation, recent studies have shown that m6A modification within multiple types of RNAs is a dynamic and reversible process. The m6A abundance depends on two pivotal proteins, namely, methyltransferases (MTs; m6A writers) and demethylases (m6A erasers). Writers and erasers are involved in adding or removing methyl groups to the conserved sequence. In addition to these two pivotal proteins, the third proteins (m6A readers) recognize specifically m6A marks and ultimately perform the specific biological functions of m6A-modified RNAs. In general, writers, erasers, and readers form a sophisticated regulatory system that guides the formation, removal, and decoding of m6A modifications.

Although many efforts have been made to investigate the m6A modification in animals, the research focusing on m6A regulatory genes in the plant kingdom is still in its infancy. Recently, major components of m6A writer complexes have been systemically identified and investigated in *Arabidopsis* (Yue et al., 2019). The addition of m6A modification is not controlled by a single RNA MT, but by a protein complex formed by a series of m6A writer components (Śledź et al., 2016). As the first m6A writer component discovered in plants, the MT family can be sorted into three subfamilies, namely, methyltransferase A (MTA), methyltransferase B (MTB), and methyltransferase C (MTC). MTA contained a catalytic core domain (MT-A70) that exerted RNA modification activity. Subsequently, the other MT member, MTB, was identified and considered to be the second most-active enzyme in regulating m6A level. In addition, previous research has shown that MTB not only has a supporting role in interacting with MTA to form a stable MTA–MTB heterodimer but also plays a crucial role in binding m6A writer complexes to RNA substrates (Wang et al., 2016a; Knuckles et al., 2018). As a newly discovered subfamily of MT family, there are few studies on MTC and its specific role in m6A modification (Liang et al., 2020). More research is needed to determine the role of MTC in m6A writing. It has not been proposed whether MTC is also involved in the formation and maintenance of MTA–MTB heterodimer. Except for the aforementioned MT family, the composition of m6A writer complex also required a series of other important components, including FKBP interacting protein 37 (FIP37), virilizer (VIR), and E3 ubiquitin ligase HAKAI. Among them, FIP37 was considered as an ortholog of Wilms’ tumor 1-associating protein (WTAP). Inhibition of FIP37 expression was found to significantly reduce the overall m6A level (Shen et al., 2016). It has shown that FIP37 also plays an indispensable role in sculpting m6A modification. The third core component of the m6A writer complex, VIR, is known for guiding the MTA–MTB heterodimer to the target region of specific RNAs. Similar to the function of FIP37, downregulation of HAKAI expression can cause a significant decrease in relative m6A levels (Růžička et al., 2017). Additionally, it has been found that HAKAI can interact with other m6A writer complex members and was required for RNA methylation.

The level of RNA methylation in plants is not only regulated by m6A writers, but also by m6A erasers. Previous studies have confirmed that m6A marks are removed by α-ketoglutarate-dependent dioxygenase homolog (ALKBH) that can wipe methyl groups off m6A-modified RNAs and restore the m6A to unmethylated adenosine (Scarrow et al., 2020). In contrast to RNA methylation that is catalyzed by an m6A write complex, m6A demethylation process required only a single m6A demethylase family. ALKBH, which contained a conserved clavaminate synthase-like domain, has catalytic activity and can effectively reverse m6A marks. The high abundance of ALKBH in *Arabidopsis thaliana* and *Solanum lycopersicum* was correlated with the decrease in the relative m6A level (Martínez-Pérez et al., 2017; Zhou et al., 2019a). However, the functional roles of m6A eraser in species outside of the *Arabidopsis* and tomato need to be further discovered. Besides, decoding of m6A marks was tightly bound up with the implementation of m6A biological function, which required m6A reader to recognize m6A-modified sites and perform varying functions during mRNA processing. Two classes of m6A readers have been reported, namely, evolutionarily conserved C-terminal region (ECT) and cleavage and polyadenylation specificity factor 30 (CPSF30; Addepalli and Hunt, 2007; Arribas-Hernández et al., 2018). ECT containing the conserved YTH domain can specifically recognize m6A marks through a canonical aromatic cage. Moreover, the absence of ECT results in the loss of m6A binding affinity, which is similar to the phenomenon caused by the knockdown of *MTA* expression (Scutenaire et al., 2018). As another m6A reader, CPSF30, contained a highly conserved C3H1-type zinc finger domain at N-terminal region, which was mainly responsible for scavenging deleterious mRNA transcripts through m6A-assisted polyadenylation processing (Pontier et al., 2019).

To date, existing reports clearly demonstrate that RNA methylation plays a prominent role in response to diverse environmental stresses, including drought (Scutenaire et al., 2018; Miao et al., 2020), cold (Hu et al., 2019), and UV radiation stresses (Dominissini et al., 2012). Under external stress, the increase of m6A marks in the 5' untranslated region (UTR) promoted the translation of drought-resistant mRNA (Meyer et al., 2015). Meanwhile, m6A RNA methylation can also alleviate the damage of abiotic stress *via* modulating mRNA abundance, splicing, stability, and decay (Zhou et al., 2015, 2018; Duan et al., 2017; Kramer et al., 2020). Additionally, recent studies have shown that the dynamic redistribution of m6A levels under stress leads to m6A marks mainly enriched in genes related to primary and secondary metabolisms (Liu et al., 2020). At present, the major knowledge on the biological function and regulatory mechanism of m6A has been limited to a handful of model plant taxa. There is still a significant gap surrounding m6A-mediated regulatory mechanism in crop plants, especially horticultural plants.

Tea plant (*Camellia sinensis*), which originated in southwest China, is one of the most economically important crop plants around the world. During the lifespan of tea plants, tea yield and quality are tightly linked to the environmental condition. With the more frequent occurrence of extreme climate, sustainable development of the global tea industry is also continuously threatened by a multitude of external stresses, particularly extreme cold and drought. Increasing studies have focused on molecular mechanisms of tea plants underlying the stress response (Wang et al., 2016b; Guo et al., 2017; Shen et al., 2019a; Zhang et al., 2020a). An array of stress-induced genes and core transcription factors have been identified in tea plants, and they play vital roles in coping with multiple stresses (Zhu et al., 2018a; Shen et al., 2019b; Zhou et al., 2019b; Li et al., 2020a; Wang et al., 2020). Additionally, oolong tea, one of the Chinese premium tea processes, is well-known for its mellow taste and elegant floral-fruity fragrance. The unique flavor of oolong tea is closely linked to its manufacturing process. Reportedly, withering is the first indispensable step to improve the quality of oolong tea during postharvest manufacturing process (Hu et al., 2018; Wang et al., 2019). Similar to preharvest tea leaves, postharvest leaves were also exposed to various environmental stresses during tea-withering stage. Moreover, it is reported that plants respond to environmental stress through DNA methylation and histone methylation modifications. More recently, DNA methylation and histone methylation-related regulatory genes have been investigated and found to play pivotal functions in stress response (Zhu et al., 2020; Chen et al., 2021). However, the roles of RNA methylation and related regulatory genes in tea plants against various stresses during preharvest and postharvest processing are still far from clear to date. The chromosome-scale genome of tea plants provides an opportunity to accurately investigate m6A regulatory genes and systemically dissect the potential functions of RNA methylation in tea plants during preharvest and postharvest processing. Here, we initially identified and classified the m6A regulatory genes from tea plants and analyzed their evolutionary relationships, chromosomal distribution, and gene structure at the whole genome scale. Then, we examined the expression profiles of m6A regulatory genes under abiotic stresses and withering process. Finally, the relationship between m6A regulatory genes and m6A level as well as the roles of RNA methylation during preharvest and postharvest processes was explored through functional assessment. Our findings lay a foundation for exploring the diverse functions of m6A regulatory genes in tea plants under environmental stresses and highlight the underlying effects of m6A modifications on the precise regulation of tea quality during the withering stage of postharvest processing.

## Materials and Methods

### Identification and Characterization of m6A Regulatory Genes in Tea Plants

To identify all members of the m6A regulatory genes, the known sequences of m6A regulatory genes in *A. thaliana* and *S. lycopersicum* were obtained from The Arabidopsis Information Resource (TAIR; Berardini et al., 2015) and Sol Genomics Network (SGN) database (Bombarely et al., 2010), respectively. These sequences were queried against the chromosome-level genome of tea plant (Xia et al., 2020) using BLAST algorithm with the parameter E-value < 1.0E^−5^. Then, the hidden Markov model (HMM) profiles of the MT-A70 family (PF05063), WTAP family (PF17098) and virilizer domain (PF15912), 2OG-Fe(II) oxygenase superfamily (PF13532), and YTH domain were used to evaluate the deduced protein sequences of m6A regulatory genes in *C. sinensis*. The obtained sequences were further confirmed using Simple Modular Architecture Research Tool (SMART; Letunic and Bork, 2017) and Conserved Domain Database (CDD; Lu et al., 2019). The physicochemical parameters of the relevant proteins were examined using the ExPASy tool (Artimo et al., 2012). The Plant-mPLoc program (Chou and Shen, 2010) was used to analyze the potential subcellular localization of these proteins. The percent identity matrix of m6A regulatory genes was calculated using the Clustal Omega program (Li et al., 2015). The conserved domains in m6A regulatory proteins were detected using the PfamScan tool (Madeira et al., 2019).

### The Phylogenetic Classifications, Chromosomal Distributions, and Gene Structures of m6A Regulatory Genes in Tea Plants

The phylogenetic trees were generated using the neighbor-joining (NJ) algorithm with 1,000 bootstrap replicates in MEGA X software (Kumar et al., 2018) and then were visualized using the Evolview v3 server (Subramanian et al., 2019). The detailed protein sequences from the dicotyledonous species (*C. sinensis*, *A. thaliana*, *S. lycopersicum*, *Vitis vinifera*, and *Gossypium hirsutum*), monocotyledonous species (*Zea mays*, *Triticum aestivum*, and *Oryza sativa*), pteridophyte species (*Selaginella moellendorffii*), and moss species (*Marchantia polymorpha* and *Physcomitrella patens*) were listed in Supplementary Table S1.

On the basis of the genome annotation files at the Tea Plant Information Archive (TPIA) database (Xia et al., 2019), the chromosomal distributions and gene structures of m6A regulatory genes were visualized using the TBtools software (Chen et al., 2020a). The MEME suite (Bailey et al., 2009) was employed to investigate the conserved motifs in m6A regulatory proteins.

### The Protein–Protein Interactions, Evolutionary Selections, Gene Duplication Events, and Collinearity Analysis of m6A Regulatory Genes in Tea Plants

The protein–protein interaction networks of m6A regulatory proteins were constructed using the STRING v11.0 database (Szklarczyk et al., 2018). The gene duplication events were investigated using MCScanX software (Wang et al., 2012) with the default parameters. The TBtools software was used for the collinearity analysis of m6A regulatory genes. To reckon the evolutionary selections of m6A regulatory genes, the TBtools software was used to calculate the nonsynonymous substitution rate (Ka), synonymous substitution rate (Ks), and Ka/Ks ratio of each duplicated gene pair. The divergence time (T) of duplicated gene pairs was calculated according to the formula T = Ks/(2 × 6.5 × 10^−9^) × 10^−6^ million years ago (Mya; Wei et al., 2018).

### Analysis of *cis*-Acting Elements in the Promoter Regions of m6A Regulatory Genes in Tea Plants

The promoter sequences of the m6A regulatory genes, which were 2,000 bp upstream from the translation start site, were collected from the TPIA database. The obtained sequences were then submitted to the PlantCARE database for *cis*-acting element analysis (Lescot et al., 2002).

### Expression Profiles of m6A Regulatory Genes in Tea Plants Based on Transcriptome Data

Public transcriptome data, including eight representative tissues (apical buds, young leaves, mature leaves, old leaves, stems, roots, flowers, and fruits) and different withering processes (FL, fresh leaves; IW, indoor-withered leaves; SW, solar-withered leaves), were collected from the Sequence Read Archive (SRA) database (accession numbers PRJNA274203 and PRJNA562623). After quality trimming, all clean reads were mapped to the chromosome-level genome of tea plant using the HISAT software (Kim et al., 2015), and then the RSEM (Li and Dewey, 2011) was used to calculate the fragments per kilobase per million mapped reads (FPKM) value. The expression profiles of m6A regulatory genes were visualized using the TBtools software based on the normalized FPKM values.

### Plant Materials and Treatments

The tea plants (*C. sinensis* cv. Tieguanyin) were grown at Fujian Agriculture and Forestry University, Fuzhou, Fujian Province, China (E 119°14′, N 26°05′). Referring to the previous method (Zhu et al., 2020), the tea plants were subjected to abiotic stress treatments. To simulate drought treatment, tea leaves were sprayed with 15% (w/v) PEG-4000 solution. For cold treatment, the tea plants were transferred to the artificial climatic chamber, and temperature was set to 4°C. Then, all the tender leaves from treated tea plants were sampled at 0, 12, 24, 36, and 48 h, respectively. Three independent biological replicates were conducted for each sample.

For the withering process of oolong tea, the tender shoot containing one bud and first three leaves were picked uniformly from tea plants. The picked leaves were evenly divided into three parts. The first part was collected immediately as the FL. The second part was exposed to sunlight for 45 min. The third part was subjected to indoor-withering for 45 min. The detailed parameters in the solar-withering and indoor-withering were described in the previous study (Zhu et al., 2020). The FL, IW leaves, and SW leaves were sampled for further analyses. Each sample was performed in three independent biological replicates. All collected samples were immersed in liquid nitrogen immediately and then maintained at −80°C until further investigation.

### Total RNA Isolation and Relative Expression Analysis of m6A Regulatory Genes in Tea Plants

Total RNA was isolated separately from the above-mentioned samples using the Trizol reagent (TransGen Biotech, Beijing, China). The isolated RNA integrity was verified by gel electrophoresis and Agilent Bioanalyzer 2100 system (Thermo Fisher Scientific, MA, United States). Then RNA was used to synthesize the first-strand cDNA using the TransScript First-Strand cDNA Synthesis SuperMix Kit (TransGen Biotech, Beijing, China). The quantitative real-time polymerase chain reaction (qRT-PCR) of m6A regulatory genes was conducted on the LightCycler 480 platform (Roche Applied Sciences, Basel, Switzerland) according to our previous method (Zhu et al., 2020). *Glyceraldehyde-3-phosphate dehydrogenase* (*GAPDH*) and *β-actin* were used to normalize the gene expression. The relative gene expression was calculated using 2^-ΔΔCT^ method (Livak and Schmittgen, 2001). All specific primers are listed in Supplementary Table S2. Three biological replicates were performed for each qRT-PCR analysis.

### Quantitative Analyses of Global N6-Methyladenosine RNA Methylation and 5-Methylcytosine DNA Methylation

Global m6A RNA methylation level in tea leaves was determined using the EpiQuik m6A RNA Methylation Quantification Kit (Epigentek, Farmingdale, NY, United States), according to the manufacturer’s instructions. The m6A level was detected colorimetrically by reading the absorbance in the Infinite M200 PRO enzyme-labeled instrument (Tecan, Switzerland).

To evaluate the global 5mC DNA methylation level in tea leaves, genomic DNA from FL, IW, and SW was extracted separately using EasyPure Plant Genomic DNA kit (TransGen Biotech, Beijing, China). The extracted DNA was checked in gel electrophoresis and quantified by Agilent Bioanalyzer 2100 system. According to the manufacturer’s protocols as previously described (Zhou et al., 2019a), the integrated DNA was used to detect the 5mC level by MethylFlash Global DNA Methylation ELISA Easy Kit (Epigentek, Farmingdale, NY, United States). Each experiment was repeated three times independently.

### Functional Assessment of m6A Regulatory Genes in Tea Leaves by siRNA-Mediated Gene Silencing

To uncover the potential functions of m6A regulatory genes in tea leaves, gene suppression was performed as previously described (Zhao et al., 2020) with minor modifications. The freshly apical buds with first leaves were picked uniformly from eight-year-old tea plants (*C. sinensis* cv. Tieguanyin) at the development stage of one bud with three leaves. Tea shoots (the apical buds with first leaves) were then incubated in 1.5-mL microcentrifuge tubes containing 1 ml of 20 μM siRNA solution at room temperature. Shoots incubated with the same concentrations of siRNA-negative control (siRNA-NC) were used as the internal control. After 12- and 24-h incubations, the collected shoots were used for methylation level and qRT-PCR analyses. The flavonoid content of these shoots was also determined as described elsewhere (Zhu et al., 2019). The specific siRNAs targeting m6A regulatory genes were synthesized from GenePharma (Shanghai, China). Detailed information on siRNA and siRNAs-NC is provided in Supplementary Table S2.

### Statistical Analyses

All data were presented as the mean ± SD. Group differences were conducted by one-way analysis of variance followed by Tukey’s *post-hoc* test, and all statistical analyses were conducted on SPSS 25 software. The rMATS software was employed to detect the alternative splicing (AS) events (Shen et al., 2014). The correlation analysis of m6A regulatory genes, 5mC regulatory genes, m6A level, and 5mC level was performed based on Pearson’s correlation coefficient. The expression levels of 5mC regulatory genes were obtained from our previous study (Zhu et al., 2020).

## Results

### Identification and Characterization of m6A Regulatory Genes in Tea Plants

After thoroughly screened the whole genome of tea plant, a total of nine m6A writer, 16 m6A eraser, and nine m6A reader genes were ultimately ascertained. All m6A regulatory genes were named according to their homologs of *A. thaliana*. Their protein lengths and predicted molecular weights (MWs) varied widely (Supplementary Table S3). The lengths of putative m6A regulatory proteins ranged from 242 (CsALKBH2) to 1,387 (CsVIR1 and CsVIR2) amino acids, and the MW of m6A regulatory proteins was between 27.52 (CsALKBH2) and 152.50 (CsVIR1). Likewise, the m6A regulatory proteins had considerably different theoretical isoelectric point. CsALKBH3 had the highest theoretical isoelectric point (9.30), while CsALKBH5A had the lowest theoretical isoelectric point (4.77). Additionally, the subcellular localization of these proteins showed that a total of 30 m6A regulatory proteins (88.24%) were localized in the nucleus, with the remaining four proteins (CsALKBH5C, CsALKBH9, CsALKBH11, and CsECT5) targeted to the cytoplasmic region.

### The Phylogenetic Classifications and Structural Features of m6A Regulatory Genes in Tea Plants

**Figure 1:**
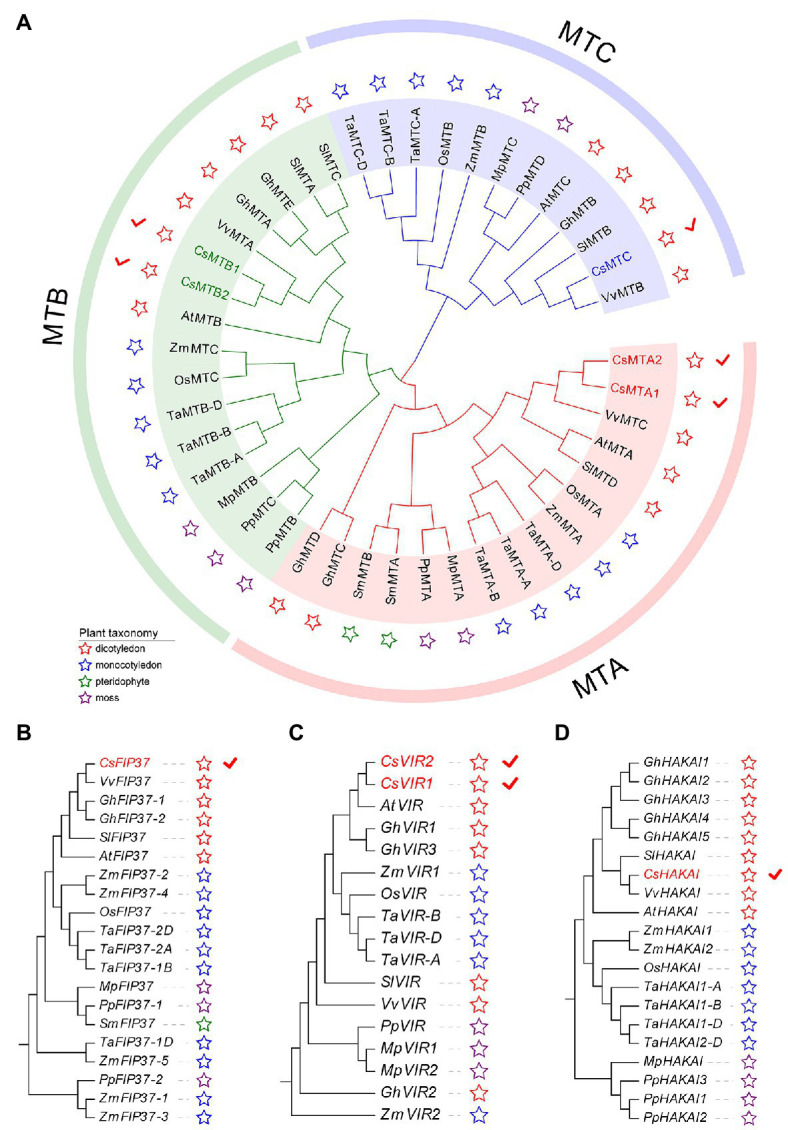
Phylogenetic analysis of m6A writer genes from five dicotyledons, three monocotyledons, one pteridophyte, and two mosses. **(A)** Phylogenetic tree of *MT* genes. **(B)** Phylogenetic tree of *FIP37* genes. **(C)** Phylogenetic tree of *VIR* genes. **(D)** Phylogenetic tree of *HAKAI* genes.

**Figure 2:**
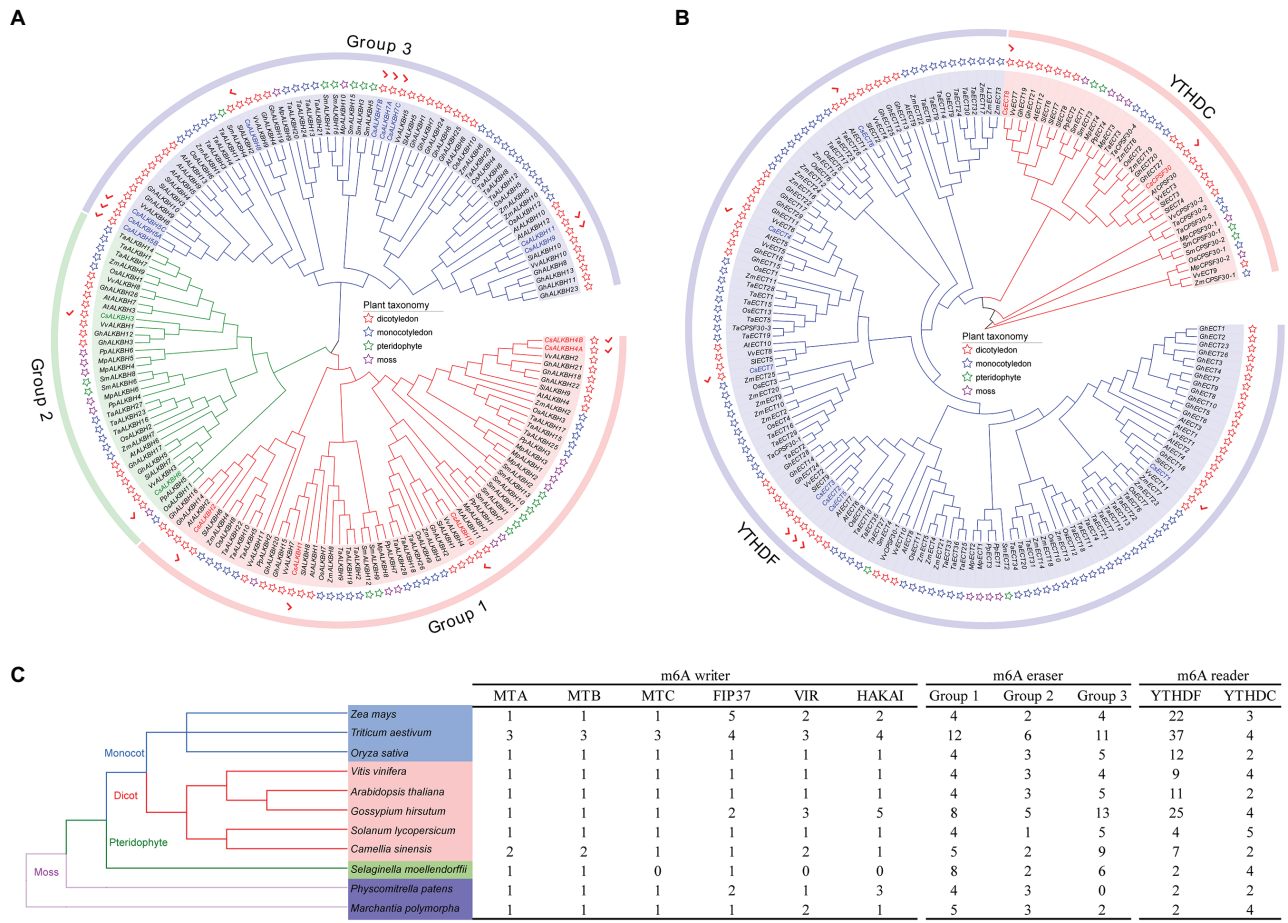
Evolutionary analysis of m6A regulatory genes from five dicotyledons, three monocotyledons, one pteridophyte, and two mosses. **(A)** Phylogenetic tree of m6A eraser genes. **(B)** Phylogenetic tree of m6A reader genes. **(C)** Number of m6A regulatory genes in different plant species.

To gain an insight into the evolutionary history of the m6A regulatory genes in the plant kingdom, the m6A writer, m6A eraser, and m6A reader proteins from five dicotyledons, three monocotyledons, one pteridophyte, and two mosses were used to construct phylogenetic trees. The m6A writer can be naturally grouped into four categories, namely, MT, FIP37, VIR, and HAKAI. As per phylogenetic analysis, the MT gene family can split into three distinct clades named as MTA, MTB, and MTC (Figure 1A). Two CsMTAs, two CsMTBs, and one CsMTC were, respectively, distributed in their corresponding evolutionary clades. The MTA, MTB, and MTC were present in all dicotyledons, monocotyledons, and mosses. However, both MTB and MTC subfamilies were absent in the pteridophyte, and only MTA subfamily was identified from this plant. Similarly, only FIP37 was detected in the pteridophyte (Figures 1B–D). However, there was an absence of VIR and HAKAI proteins from this species. On the other hand, the phylogenetic tree of ALKBH family was stringently clustered into three groups (Figure 2A). In each group, most ALKBH proteins from moss and pteridophyte lie in a distinct branch, while ALKBH proteins belonging to spermatophyte were grouped into other branches. In the phylogenetic tree of m6A readers, a total of 154 ECT and 15 CPSF30 proteins fell into two distinct clades (YTHDF and YTHDC; Figure 2B), which were similar to the previous report (Scutenaire et al., 2018). Intriguingly, most m6A reader proteins from moss were closely clustered with their homologs in pteridophyte than in spermatophyte.

Besides, the number of m6A regulatory genes varied greatly among the 11 plant species (Figure 2C). The number of m6A regulatory genes in hexaploid wheat (*T. aestivum*) and tetraploid cotton (*G. hirsutum*) was observed almost double of several plant species, including *C. sinensis* (34), *O. sativa* (32), and *A. thaliana* (32), indicating that the m6A regulatory genes in polyploid plants have exhibited large-scale expansion. Notably, a total of 34 m6A regulatory genes were screened in tea plants, which had the fourth-highest number of m6A regulatory genes among the seven plant species.

**Figure 3:**
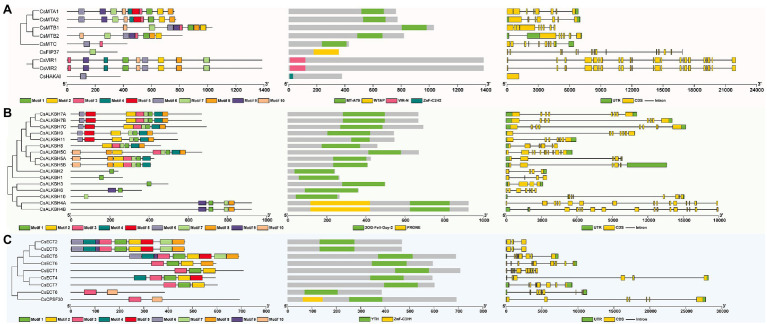
Phylogenetic relationships, conserved motifs, functional domains, and exon–intron organizations of m6A regulatory genes in tea plants. **(A)** Structure analysis of m6A writer genes. **(B)** Structure analysis of m6A eraser genes. **(C)** Structure analysis of m6A reader genes.

To analyze the sequential characteristics of m6A regulatory components from tea plants, the conserved domains and motif sequences were identified from these proteins. The typical MT-A70 domain was found in each member of CsMT family (Figure 3A). Among other m6A writer components, we found that CsFIP37 protein was conserved with one WTAP domain. On the other hand, both two CsVIR proteins contained one conserved VIR-N domain, and the CsHAKAI had a ZnF-C2H2 domain. Unlike the m6A writer components that contain diverse conserved domains, all members of ALKBH family had one 2OG-Fell-Oxy-2 domain (Figure 3B) and shared a high level of similarity with each other. It is worth noting that both CsALKBH4A and CsALKBH4B harbored one additional PRONE domain. Similarly, one highly conserved YTH domain was identified in all ECT proteins belonging to m6A readers (Figure 3C). Another key component of the m6A readers, CPSF30, possessed one extra ZnF-C3H1 domain.

Among different m6A writer components, the motif number varied from 1 to 10 (Supplementary Figure S1). CsFIP37 and CsHAKAI harbored only one motif. In contrast, homologous CsMTA1 and CsMTA2 contained all 10 motifs. With the exception of the CsALKBH10, the other members of ALKBH family had one motif 1. Further, motif 10 was found to be specific to CsECT8 and CsCPSF30 of YTHDC subfamily, while motifs 1 and 2 only existed in the YTHDF subfamily. Thereafter, the analysis of the exon–intron organizations showed that there was a large variation in the exon number of m6A regulatory genes. Both *CsVIR1* and *CsVIR2* contain up to 23 exons. Contrastingly, *CsHAKAI* was found to be intronless. In general, most m6A regulatory proteins in the adjacent branches exhibited similar motif distribution and the exon–intron compositions.

### The Chromosomal Distributions, Gene Duplication Events, and Evolutionary Selection of m6A Regulatory Genes in Tea Plants

**Figure 4:**
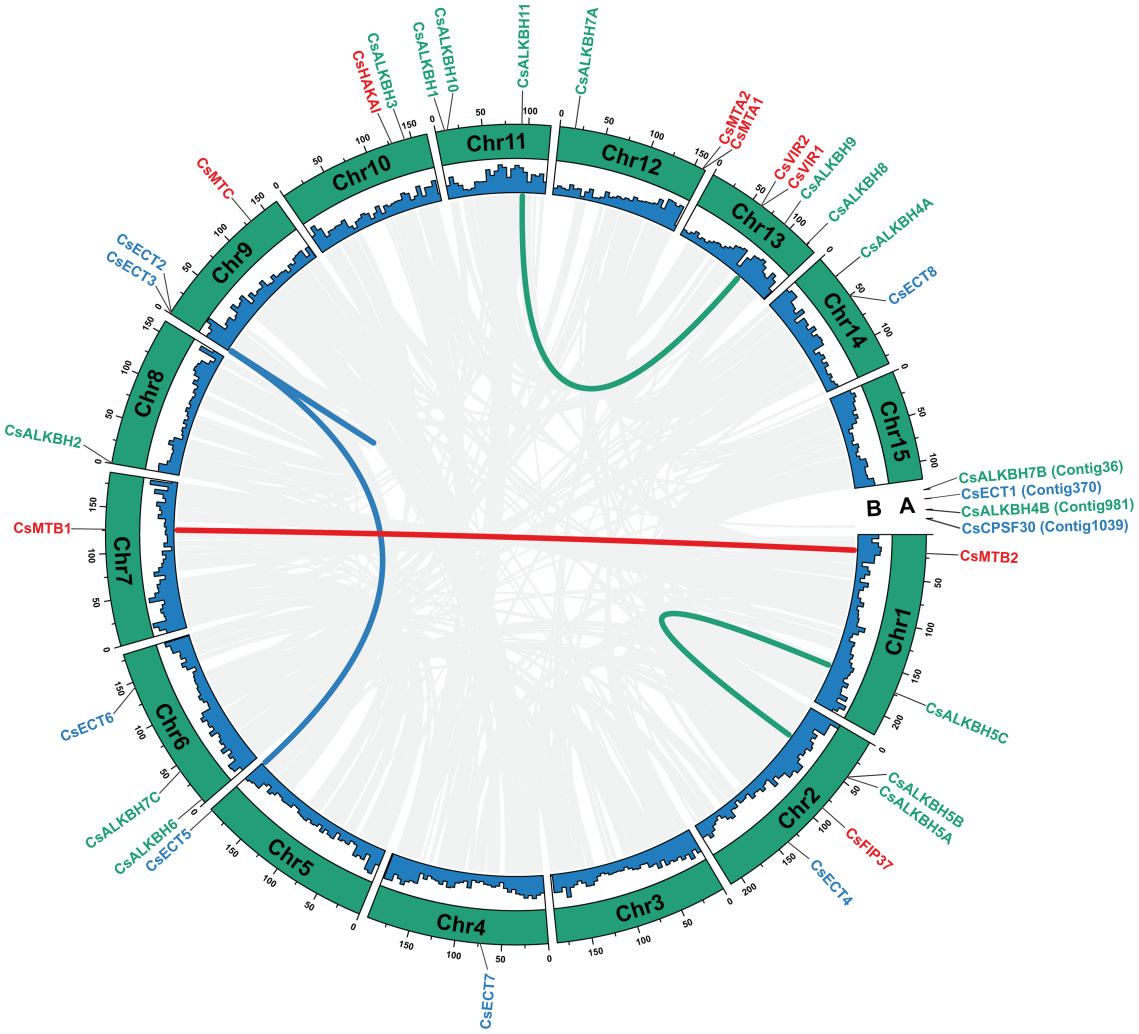
Chromosomal distribution and collinearity analysis of m6A regulatory genes in tea plants. **(A)** Chromosomal number. **(B)** GC content. The gray lines represent all collinear blocks in the genome of tea plant. The other colored lines represent all collinear relationships of m6A regulatory genes.

In total, 30 m6A regulatory genes were randomly distributed on 13 chromosomes, and the remaining four genes were located on the unanchored contigs (Figure 4). Both chromosomes 2 and 14 harbored the highest number of m6A regulatory genes (4), whereas chromosomes 4, 5, 7, and 8 contained only one gene. Besides, a total of five duplicated gene pairs involved in nine m6A regulatory genes were observed in the tea genome, namely, two m6A writer genes, four m6A eraser genes, and three m6A reader genes. Additionally, these nine m6A regulatory genes may experience the segmental duplication or whole-genome duplication (WGD) events.

**Figure 5:**
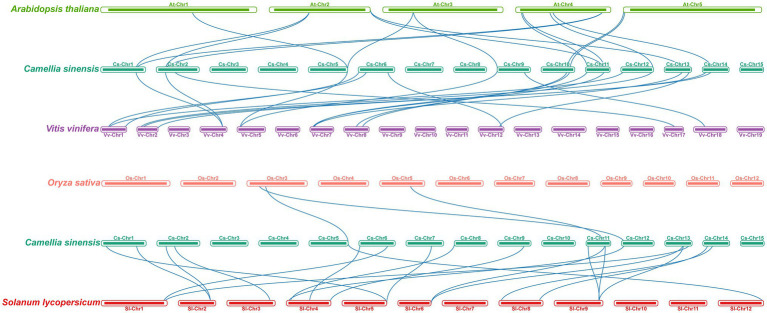
Collinearity analysis of m6A regulatory genes between tea plants and four representative plant species. The blue lines represent all collinear relationships of m6A regulatory genes between tea plants and other plants.

To further explore the evolution patterns of the m6A regulatory genes in different plant species, the comparative collinearity analyses of tea plants associated with four representative species were performed (Figure 5). A total of 24 m6A regulatory genes in tea plants exhibited the collinearity relationships with those in *V. vinifera*, followed by *S. lycopersicum* (17), *A. thaliana* (14), and *O. sativa* (3). Curiously, the number of collinearity gene decreased sharply between dicotyledons and monocotyledons. The number of homologous m6A regulatory genes between tea plants and other dicotyledons is far more than that between tea plants and monocotyledons. This result indicated that the expansion events of m6A regulatory genes may occur after the monocot–dicot divergence. In addition, four ALKBH members (CsALKBH5B1, CsALKBH5C, CsALKBH9, and CsALKBH1) and two ECT members (CsECT1 and CsECT5) were shown to have more than one pair of collinearity gene.

To explore the evolutionary trajectory acting on these m6A regulatory genes, we calculated the Ka, Ks, and, Ka/Ks ratio for each duplicated pair of m6A regulatory genes in tea plants (Table 1). The Ka/Ks ratio of five duplicated pairs of m6A regulatory genes ranged from 0.2130 to 0.3838, with an average of 0.2837, indicating that these m6A regulatory genes evolved through strong purifying selection, which limited the divergences of gene functions after segmental duplication or WGD events. Based on the previously defined criteria (Xia et al., 2020), the divergence time of duplicated gene pairs can be estimated using Ks value. In the present study, the Ks values of these five duplicated gene pairs were distributed between 0.0160 and 0.5217. Among them, the duplicated gene pair (*CsECT2*-*CsECT3*) possessed the lowest Ks value (0.0160), implying that it probably generated from the recent segmental duplication or WGD event. The Ka values of the other four duplicated gene pairs were in the range of 0.35–0.53, demonstrating that they might be associated with the ancient duplication events.

**Table 1:** The evolutionary analysis of duplicated m6A regulatory genes in tea plants.

Gene 1	Gene 2	Ka	Ks	Ka/Ks ratio	Divergence time (Mya)	Duplication type
CsMTB1	CsMTB2	0.1280	0.3573	0.3581	27.4853	WGD
CsALKBH5B	CsALKBH5C	0.1521	0.3962	0.3838	30.4747	WGD
CsALKBH9	CsALKBH11	0.1111	0.5217	0.2130	40.1309	WGD
CsECT2	CsECT3	0.0037	0.0160	0.2308	1.2288	Segmental duplication
CsECT3	CsECT5	0.0895	0.3846	0.2327	29.5868	WGD

WGD: whole-genome duplication.

### The Protein–Protein Interaction Network of the m6A Regulatory Proteins in Tea Plants

**Figure 6:**
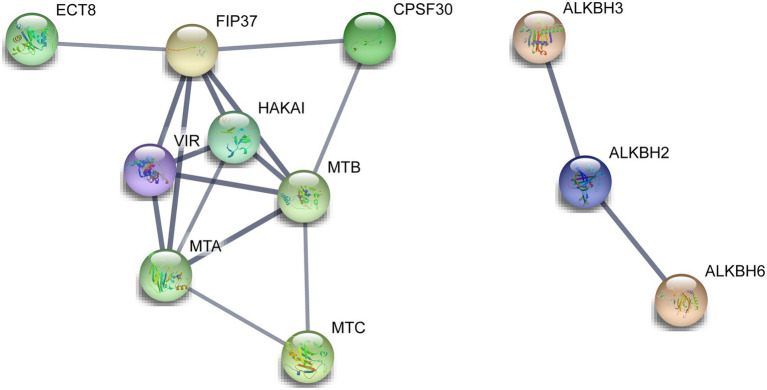
Functional protein–protein interaction network of the m6A regulatory proteins in tea plants. The protein–protein interaction network of m6A regulatory proteins was constructed using the STRING v11.0 database. The orthologous protein was found using Tea Plant Information Archive (TPIA) database, and further, the homologous proteins were also considered as STRING proteins based on the high bit score.

A total of 14 m6A regulatory proteins were aligned to the corresponding homologs of *A. thaliana*, namely, nine m6A writer components, three m6A eraser components, and two m6A reader components (Figure 6). There were strong interactions of all m6A writer components, indicating that they may be participated in m6A modifications by forming protein complexes. Meanwhile, two m6A reader components (ECT8 and CPSF30) interacted with FIP37 and HAKAI in m6A writer components. This result suggested that ECT8 and CPSF30 may immediately bind m6A sites and play ultimate regulatory roles for modified RNA. In addition, we found that ALKBH2 simultaneously interacted with the other two ALKBH members (ALKBH3 and ALKBH6), which might be associated with their special roles in the removal of m6A modification and reduction of the overall m6A level.

### Detection of *cis*-Acting Elements in the Promoter Regions of m6A Regulatory Genes in Tea Plants

**Figure 7:**
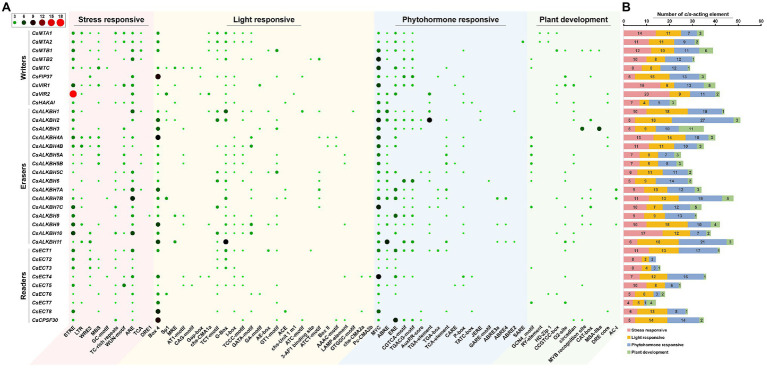
Analysis of *cis*-acting elements in the promoter regions of m6A regulatory genes in tea plants. **(A)** The distribution of *cis*-acting elements in the promoter regions of m6A regulatory genes. **(B)** The histogram of *cis*-acting elements in each category.

To better understand the transcriptional regulation and potential biological functions of m6A regulatory genes from tea plants, the *cis*-acting elements in the 2,000-bp promoter regions of 34 m6A regulatory genes were analyzed using the PlantCare database (Figure 7). In m6A regulatory components, stress-responsive, light-responsive, and phytohormone-responsive elements are more common than plant growth and development-related elements. In stress-responsive category, several types of stress-responsive elements were identified in the promoter regions of m6A regulatory genes. With the exception of *CsFIP37* and *CsALKBH6*, other 32 m6A regulatory genes contained the STRE element. Contrastingly, GC-motif element was observed in only three m6A regulatory genes (*CsMTC*, *CsALKBH7B*, and *CsECT4*). Differences in stress-responsive element types may be associated with the different functions of these genes under environmental stresses. Moreover, light-responsive elements were evenly present in the promoter regions of all m6A regulatory genes, indicating that the expression levels of m6A regulatory genes might be regulated by light signaling.

### Tissue-Specific Expression of m6A Regulatory Genes in Tea Plants

**Figure 8:**
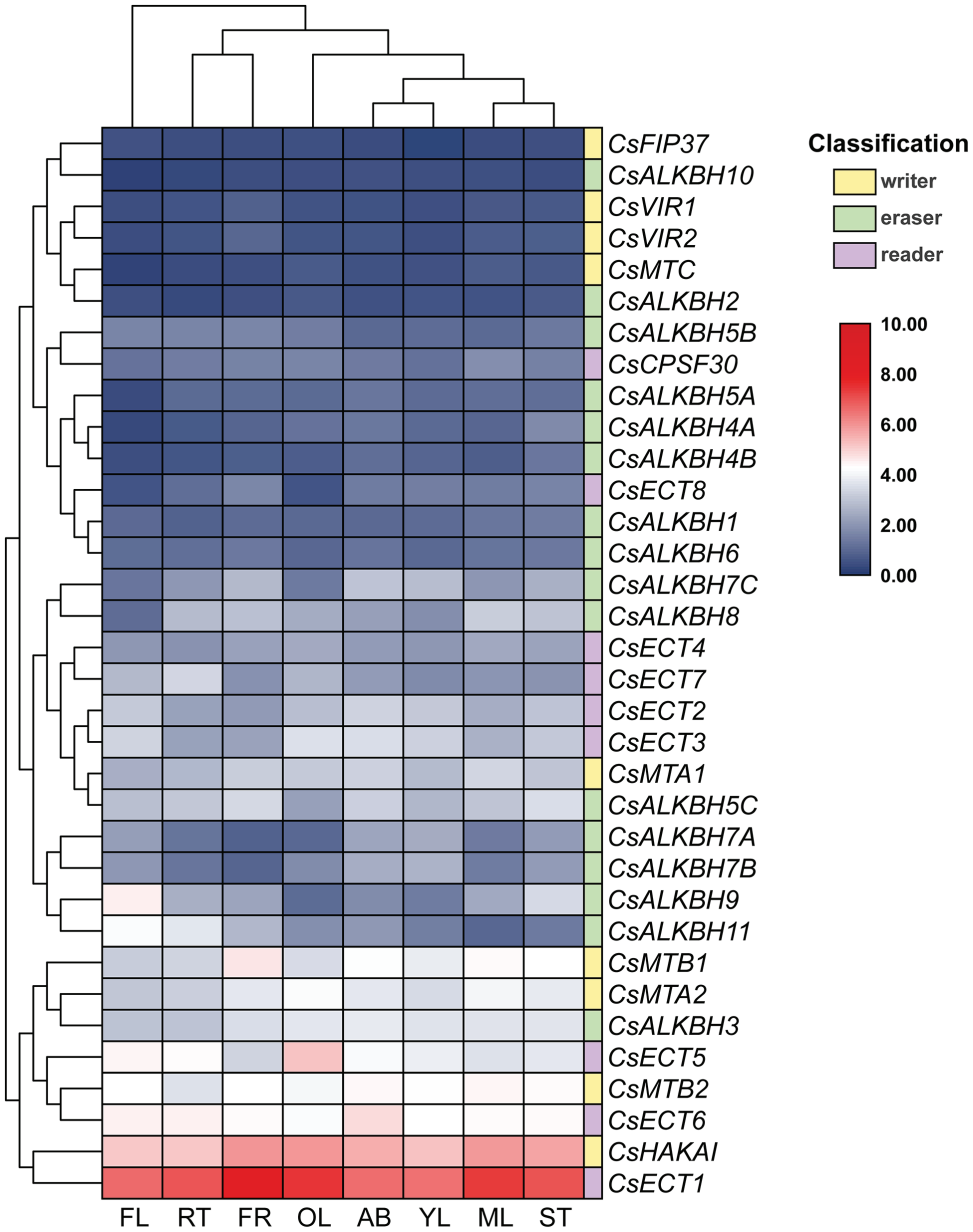
Expression profiles of m6A regulatory genes in eight tea plant tissues. The expression values of m6A regulatory genes were normalized by log_2_-transformed (FPKM+1). FL: flowers; RT: roots; FR: fruits; OL: old leaves; AB: apical buds; YL: young leaves; ML: mature leaves; ST: stems. Public transcriptome data were collected from the NCBI Sequence Read Archive (SRA) database under accession number PRJNA274203.

The expression profiles of m6A regulatory genes in eight representative tea plant tissues were retrieved from published transcriptome datasets (Figure 8). Among nine m6A writer genes, we discovered that a few m6A regulatory genes showed high expression in all studied tissues (FPKM > 10). On the contrary, the major m6A regulatory genes were seldom expressed (FPKM < 1) or expressed at moderate levels (FPKM > 1 and FPKM < 10) in eight representative tissues. It is noteworthy that *CsMTB2*, *CsHAKAI*, *CsECT1*, and *CsECT6* were continuously expressed at high levels in all tissues, implying that these m6A regulatory genes inevitably play important roles in numerous tissues. Besides, most m6A writer genes exhibited lower expression levels in young leaves than in mature leaves and old leaves. In contrast, a multitude of m6A eraser genes were more highly expressed in young leaves than in mature leaves and old leaves.

### Expression Patterns of m6A Regulatory Genes in Tea Plants Under Environmental Stresses

**Figure 9:**
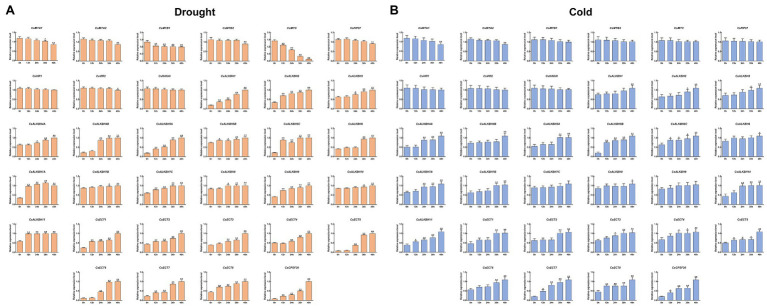
Expression patterns of m6A regulatory genes under abiotic stresses determined by qRT-PCR. **(A)** Drought stress; **(B)** Cold stress. Data are presented as mean ± SD. Single asterisk indicates significant difference (*p* < 0.05); double asterisks indicate highly significant difference (*p* < 0.01). The stress treatment was performed on the healthy two-year-old tea plants (*C. sinensis* cv. Tieguanyin) at the development stage of one bud with three leaves. To simulate drought treatment, tea leaves were sprayed with 15% (w/v) PEG-4000 solution. For cold treatment, the tea plants were transferred to the artificial climatic chamber and the temperature was set to 4°C. Then, all the tender leaves from treated tea plants were sampled at 0, 12, 24, 36, and 48 h, respectively. Three independent biological replicates were conducted for each sample.

To unveil the possible functions of m6A regulatory genes in response to environmental stresses, the expression patterns of m6A regulatory genes under drought and cold stresses were detected (Figure 9). All m6A writer genes were slightly downregulated under drought treatment. Among them, the expression levels of *CsMTB1* and *CsMTC* decreased sharply from 0 to 12 h, and then gradually decreased to the lowest level after 48-h treatment. In addition, *CsMTA1*, *CsMTA2*, *CsMTB2*, and *CsVIR2* showed no significant expression changes from 0 to 24 h but were significantly downregulated during the late stage (36–48 h) of drought stress. Remarkably, the remaining m6A genes showed no significant changes in transcript levels, suggesting that these genes might not perform the important roles under drought stress. Unlike the transcript patterns of m6A writer genes, almost all m6A eraser genes were markedly elevated with prolonged drought stress. Nine m6A eraser genes were rapidly upregulated under drought stress, but the expression levels of the other seven m6A eraser genes increased slightly at the early stage (0–24 h) of drought stress and increased significantly at 36 h after drought treatment. Similarly, the transcript levels of most m6A reader genes increased continuously and peaked at 48 h after drought treatment.

The expression patterns of all m6A regulatory genes under cold stress were similar to those under drought stress. In m6A writer genes, only *CsMTA1* and *CsMTA2* were markedly downregulated at 48 h after cold treatment, and the transcript levels of the seven m6A writer genes were not significantly altered during the whole periods of cold stress. With the exception of *CsALKBH7* and *CsALKBH9*, other m6A eraser genes were upregulated and persisted at high expression levels during the late period of cold stress. Similarly, we noted that the transcript abundance of most m6A reader genes was dramatically induced under cold stress. Four genes (*CsECT5*, *CsECT7*, *CsECT8,* and *CsCPSF30*) were upregulated immediately at 24 h, whereas the expression levels of remaining m6A reader genes were not significantly altered at the early period of cold treatments and then notably increased with prolonged cold stress.

### Effects of Withering Process on the Expression of m6A Regulatory Genes and Correlation Analyses of m6A Regulatory Genes, 5mC Regulatory Genes, m6A Levels, and 5mC Levels

**Figure 10:**
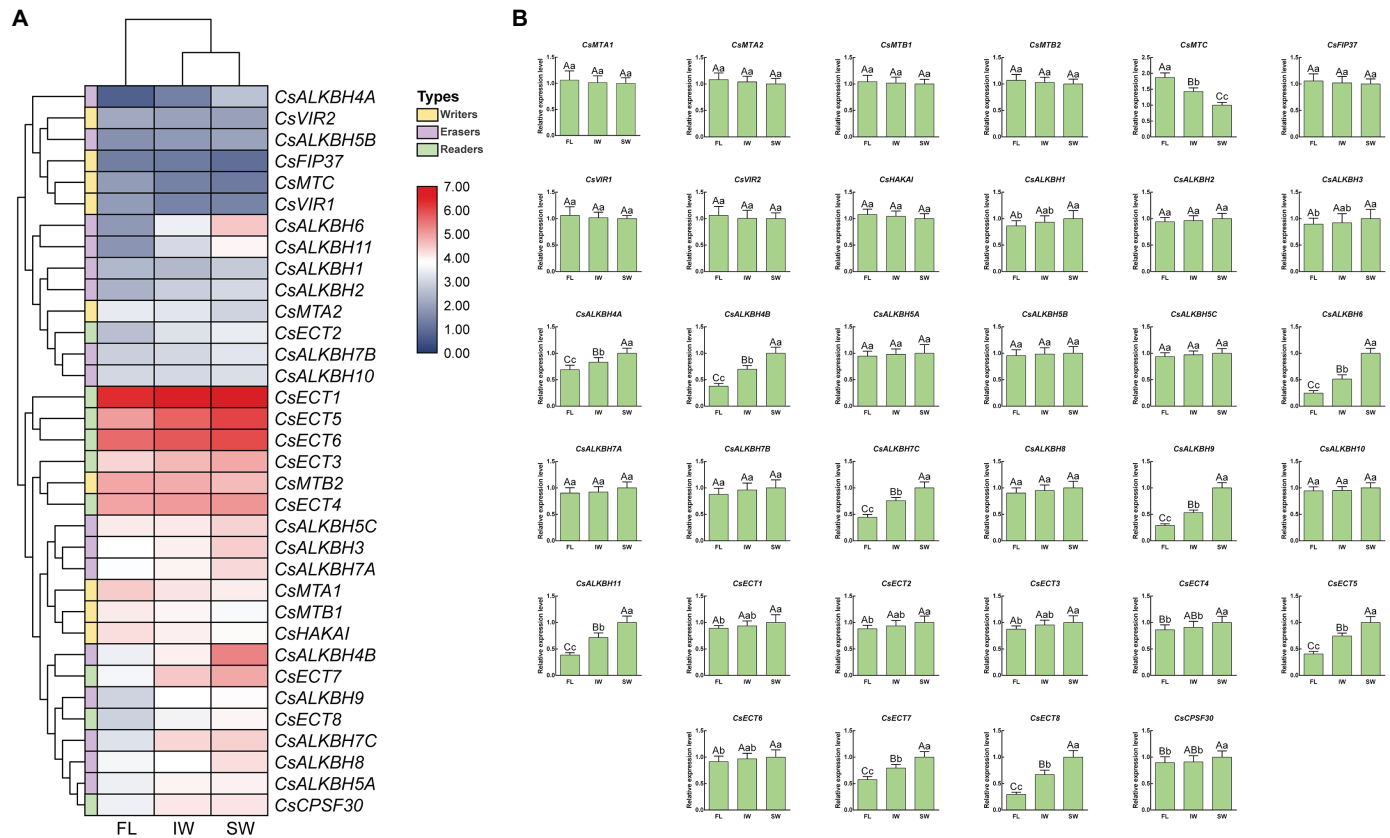
Expression patterns of m6A regulatory genes during tea-withering. **(A)** The heat map of m6A regulatory genes during tea-withering based on the transcriptome data. The expression values of m6A regulatory genes were normalized by log_2_-transformed (FPKM + 1). **(B)** Expression patterns of m6A regulatory genes during tea-withering determined by qRT-PCR. Data are presented as mean ± SD. Lowercase letter indicates significant difference (*p* < 0.05); uppercase letter indicates highly significant difference (*p* < 0.01). FL: fresh leaves; IW: indoor-withered leaves; SW: solar-withered leaves. Public transcriptome data were collected from the NCBI Sequence Read Archive (SRA) database under accession number PRJNA562623. The freshly apical buds with first leaves were picked uniformly from eight-year-old tea plants (*C. sinensis* cv. Tieguanyin) at the development stage of one bud with three leaves. The picked leaves were evenly divided into three parts. The first part was collected immediately as the FL. The second part was exposed to sunlight for 45 min. The third part was subjected to indoor-withering for 45 min. The FL, IW, and SW were sampled, respectively. Each sample was performed in three independent biological replicates.

In this study, the transcript levels of 34 m6A regulatory genes were analyzed in the FL, IW, and SW based on the previous transcriptome datasets (Figure 10A). The expression of most m6A writer genes was not affected by indoor-withering and solar-withering, while the transcript abundance of *CsMTC* was strongly reduced at IW and SW. Regarding the expression of m6A eraser genes, half of m6A eraser genes were insensitive after withering treatments, while the other eight m6A eraser genes were significantly upregulated at IW or SW. Among these eight genes, *CsALKBH1* and *CsALKBH3* were not significantly altered by indoor-withering, while the expression levels of these genes were dramatically elevated at SW. In contrast, the transcript levels of the other six *CsALKBH* genes were noticeably enhanced in IW and SW than in FL. In m6A reader genes, the expression levels of *CsECT5*, *CsECT7*, and *CsECT8* were signally higher in IW than in FL. Furthermore, the transcript abundance of all m6A reader genes was substantially upregulated and maintained at a highest level after solar-withering. These results showed that the expression levels of major m6A regulatory genes were more affected by solar-withering than by indoor-withering. Notably, we observed that a large number of alternative spliced isoforms were detected after withering treatment (Table 2), suggesting withering process may promote the occurrence of AS events. To further validate the expression patterns of all m6A regulatory genes retrieved from the transcriptome datasets, we conducted qRT-PCR analyses to determine the expression levels of these genes (Figure 10B). The overall transcript levels of the detected m6A regulatory genes were consistent with those obtained from the transcriptome datasets.

**Table 2:** Statistics of alternative splicing events at fresh leaves (FL), indoor-withered leaves (IW), and solar-withered leaves (SW).

Sample	Alternative 5' splice site	Alternative 3' splice site	Mutually exclusive exon	Retained intron	Skipped exon	Total
FL	818	1,469	347	1,411	6,047	10,092
IW	797	1,404	403	1,343	6,387	10,334
SW	856	1,501	449	1,481	7,159	11,446

**Figure 11:**
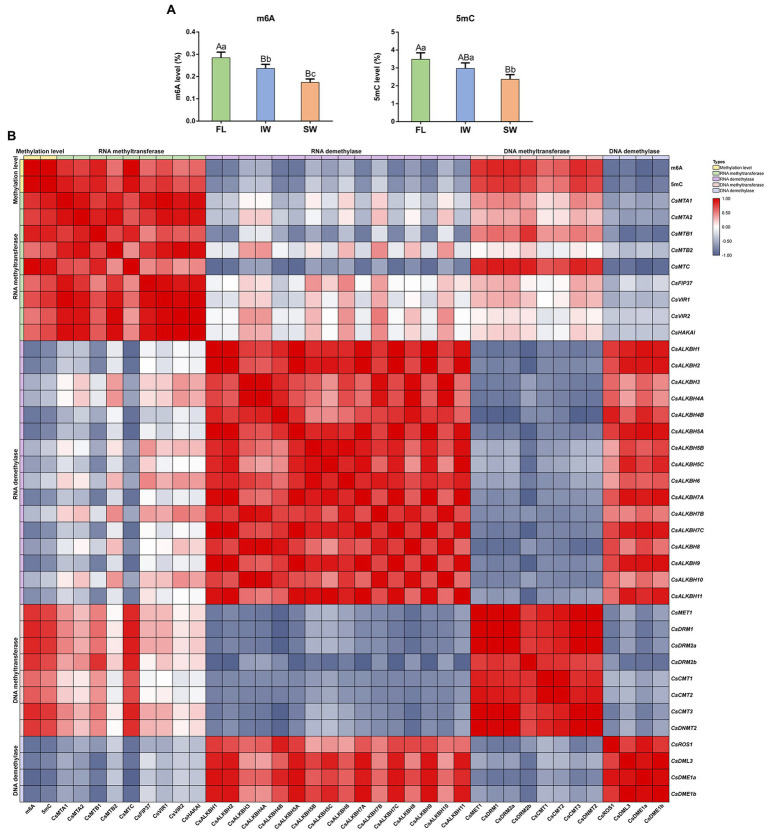
Dynamics of RNA methylation (m6A) and DNA methylation (5mC) in fresh leaves (FL), indoor-withered leaves (IW), and solar-withered leaves (SW) and correlation analyses of m6A regulatory genes, 5mC regulatory genes, m6A levels, and 5mC levels. **(A)** The levels of m6A and 5mC. **(B)** Correlation analyses of m6A level, 5mC level, and corresponding regulatory gene expression. Data are presented as mean ± SD. Lowercase letter indicates significant difference (*p* < 0.05); uppercase letter indicates highly significant difference (*p* < 0.01). To comprehensively ascertain whether there may exist the interplay between RNA and DNA methylation during tea-withering, the correlation between methylation level and corresponding regulatory gene expression was evaluated by Pearson’s correlation coefficient. Pearson’s correlation coefficient was determined by SPSS 25 software. A correlation coefficient greater than 0 indicates a positive correlation, while a correlation coefficient less than 0 indicates a negative correlation.

To comprehensively ascertain whether there may exist the interplay between RNA and DNA methylation, we examined the m6A and 5mC levels during tea-withering process. The overall m6A levels were 0.287, 0.236, and 0.173% in the FL, IW, and SW, respectively (Figure 11A). The results showed that the overall m6A level was declined after tea-withering process. We then assessed the 5mC levels in the same samples. Similarly, the 5mC level was significantly higher in FL (3.480%) and IW (2.975%) than in SW (2.363%). These results showed that the dynamic changes of m6A levels were similar to those of 5mC levels during withering process. Thereafter, we investigated the functional relationship between methylation levels and corresponding regulatory genes during tea-withering process. For these analyses, correlation networks among the expression profiles of the identified m6A regulatory genes and 5mC regulatory genes, as well as the m6A levels and 5mC levels, were conducted (Figure 11B). The m6A modifications were strongly positively correlated with the transcript abundance of RNA MT genes, but significantly negatively correlated with the transcript abundance of RNA demethylase genes. In 5mC modifications, we also detected a strong positive interaction between 5mC levels and the transcript abundance of the corresponding DNA MT genes. Contrastingly, the DNA methylation status was negatively correlated with the expression levels of DNA demethylase genes. Additionally, we found that several RNA MT genes were positively correlated with DNA MT genes. Likewise, positive correlations were detected between some RNA demethylase genes and DNA demethylase genes.

### Functional Assessment of m6A Regulatory Genes in Tea Plants

**Figure 12:**
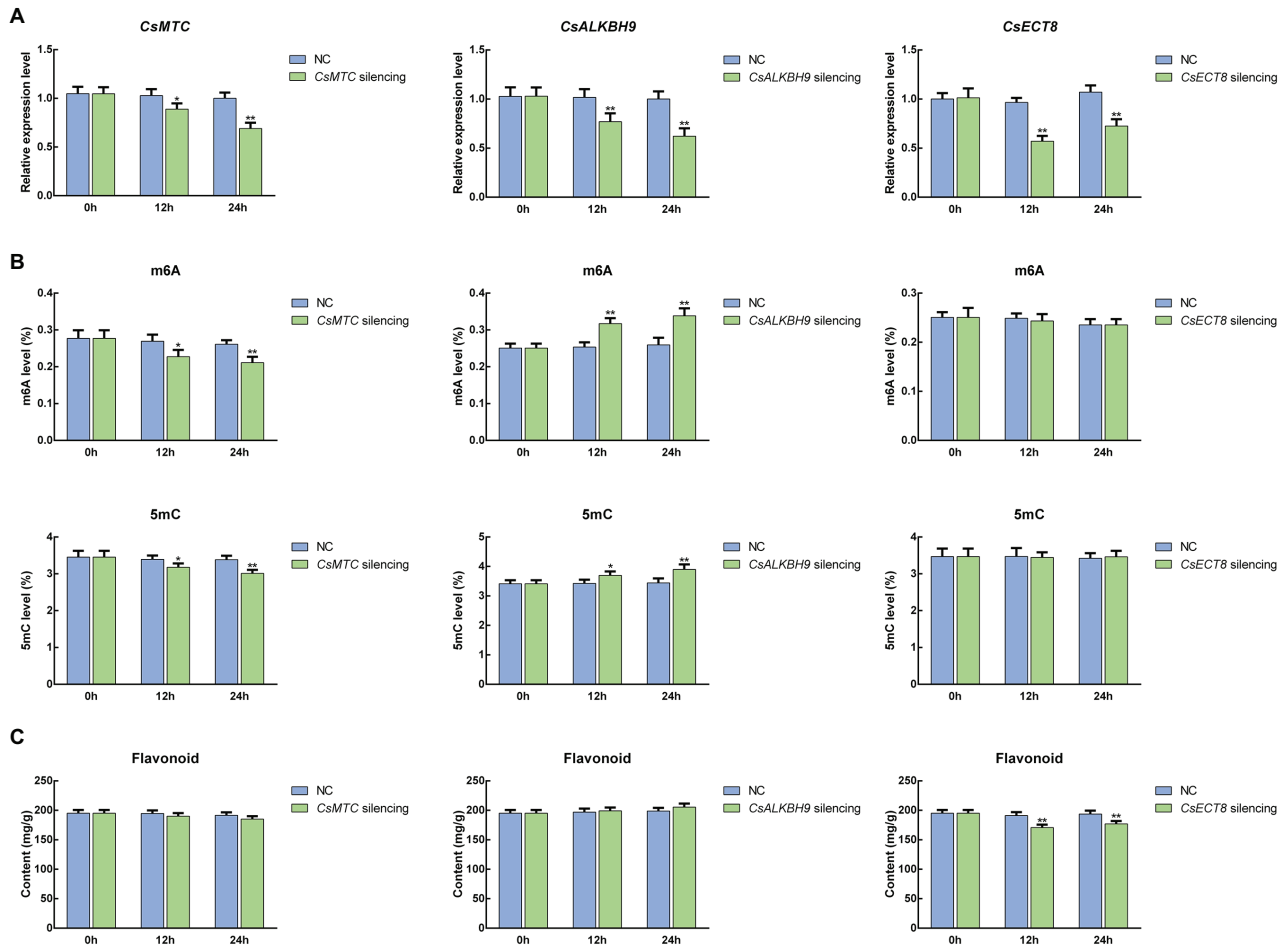
Functional assessment of m6A regulatory genes in tea leaves by siRNA-mediated transient gene silencing. **(A)** Expression patterns of m6A regulatory genes in siRNA-treated tea leaves. **(B)** The levels of RNA methylation (m6A) and DNA methylation (5mC) in siRNA-treated tea leaves. **(C)** Flavonoid content in siRNA-treated tea leaves. Data are presented as mean ± SD. Single asterisk indicates significant difference (*p* < 0.05); double asterisks indicate highly significant difference (*p* < 0.01). The freshly apical buds with first leaves were picked uniformly from eight-year-old tea plants (*C. sinensis* cv. Tieguanyin) at the development stage of one bud with three leaves. Tea shoots (the apical buds with first leaves) were then incubated in 1.5-ml microcentrifuge tubes containing 1 ml of 20 μM siRNA solution at room temperature. Shoots incubated with the same concentrations of siRNA-negative control (siRNA-NC) were used as the internal control. After 12- and 24-h incubations, the collected shoots were used for RNA isolation and flavonoid extraction.

To further unravel the potential roles of m6A regulatory genes in tea plants, the representative m6A writer gene (*CsMTC*), m6A eraser gene (*CsALKBH9*), and m6A reader gene (*CsECT8*) were selected for functional assessment *via* an siRNA-mediated silencing method. After *CsMTC-*silenced treatment, the expression level of *CsMTC* at 12 and 24 h was considerably declined compared with that at 0 h (Figure 12A). As expected, both *CsALKBH9* and *CsECT8* were also significantly downregulated at 12 and 24 h after corresponding siRNA treatments. In contrast, the transcript levels of these three m6A regulatory genes in tea leaves treated with siRNA-NC were not obviously altered. Then, the m6A level, 5mC level, and flavonoid content in gene-silenced tea leaves were also determined (Figures 12B,C). The m6A and 5mC levels were markedly declined after siRNA-*CsMTC* treatment, while the m6A and 5mC levels in tea level treated with siRNA-*CsALKBH9* were both sharply increased. However, the downregulation of *CsECT8* did not affect m6A level and 5mC levels. In addition, the flavonoid content was also significantly changed after siRNA-*CsECT8* treatment, whereas no obvious alterations of flavonoid contents in *CsMTC*- and *CsALKBH9*-silenced tea leaves were detected.

## Discussion

### Evolutionary Relationships and Structural Features of m6A Regulatory Genes in Tea Plants

In the current study, a total of 34 m6A regulatory genes were ultimately identified from the chromosome-scale genome of tea plants. Compared with other plant species, the number of m6A regulatory genes in tea plants appears greater than that in *A. thaliana* (32), *O. sativa* (32), *V. vinifera* (30), *S. lycopersicum* (26), *S. moellendorffii* (25), *M. polymorpha* (23), and *P. patens* (20). Since the genome size of tea plants (2.94 Gb) is almost several-fold larger than that of these plant species, the increasing number of m6A regulatory genes may be linked with a series of gene duplication and expansion events during the long-term evolutionary process of tea plants (Xia et al., 2020). Similarly, hexaploid wheat possessed the largest number of m6A regulatory genes, which may be attributed to the large and complex genome size (17 Gb) that has undergone polyploidization. Because the genome size of upland cotton and maize was larger than that of tea plant, more m6A regulatory genes were identified in upland cotton and maize than in tea plants, indicating that m6A modification in these two plant species may require more m6A regulatory genes to form a precise regulatory mechanism.

In phylogenetic trees of these plant taxa, the majority of m6A regulatory genes from pteridophyte and moss tend to form independent branches (Figures 1, 2), while those genes from monocotyledon and dicotyledon were generally clustered together. It suggested that these genes may have experienced expansion events after the divergence of moss and spermatophyte. Remarkably, *VIR* and *HAKAI* genes were not identified from pteridophyte. We speculated that the loss of *VIR* and *HAKAI* in pteridophyte might be due to other writer genes taking over the biological functions of these two writer genes or another alternative mechanism for RNA methylation. Gene duplication portrayed a major driving force for producing a large number of coding gene families (Defoort et al., 2019). In fact, a total of 24 m6A regulatory genes in tea plants had homologous copies in several representative dicotyledons and monocotyledons. More homologous m6A regulatory gene pairs were detected in two dicots than in monocot–dicot. This indicated that the biological functions of m6A regulatory genes may enrich and diversify after monocot–dicot divergence, and more neofunctionalization in m6A regulatory genes required to participate in this precise mechanism to cope with more complex and changeable environments during the long-term evolutionary course. Furthermore, there are five duplicated gene pairs involved in m6A regulatory genes of tea plants (Figure 4). All these duplicated gene were demonstrated to be formed after segmental duplication or WGD events. Combined with previously reported WGD events in tea genomes (Wei et al., 2018), four duplicated gene pairs were found to have experienced the WGD events, while only *CsECT2-CsECT3* gene pair underwent segmental duplication. Notably, no tandem duplicated gene pairs were detected from m6A regulatory genes. Thus, the expansion of m6A regulatory genes in tea plants was driven mainly by WGD and segmental duplication, rather than tandem duplication. In addition, duplicated event was a well-known precursor to the functional diversity of gene family, and it can confer genes with new roles in plant growth and resistance against the environmental stresses *via* neofunctionalization and subfunctionalization. The predominant traces of gene functional differentiation were tightly associated with the variation of gene sequence, structure, and expression profile (Tian et al., 2020). The Ka/Ks ratios of all duplicated m6A regulatory gene pairs were far less than 1.0 (Table 1), confirming that these m6A regulatory genes obtained from WGD or segmental duplication have experienced strong purifying selection to maintain the new acquisition of vital traits. It reflected that these duplicated genes may be conserved in the composition of their domains and motifs. Also, the functional differentiation of these genes after WGD or segmental duplication might be not conducive to the modification of RNA methylation in tea plants. In actuality, both two members of each duplicated pair were found to possess the same domains and conserved motifs (Figure 3). However, there were especially obvious differences in exon–intron structures of five duplicated gene pairs. These results clearly demonstrated that instead of a variation in conserved domain and motif, the gain or loss of exons and introns in gene sequence may contribute to the functional diversification of m6A regulatory genes. Gene expression profiling usually provided a useful indicator for dissecting its biological roles (Sun et al., 2020). In the duplicated gene pair of *CsMTB1* and *CsMTB2*, the *CsMTB1* was highly expressed in stem and root, whereas its low expression was found in flower and young leaf. The difference was that *CsMTB2* displayed a higher expression in young leaves and flower buds. Meanwhile, the lower expression of *CsMTB2* was observed in the root. In essence, the newly duplicated m6A regulatory genes and their ancestral genes tend to have divergent spatiotemporal expression profiles, which were consistent with previous results of gene families from *A. thaliana* (Zhang et al., 2020b). This indicated that the newly duplicated *CsMTB2* obtained unique functions that complemented the original functions of ancestral gene *CsMTB1* through subfunctionalization. Subsequently, we observed the expression patterns of these duplicated genes under abiotic stresses and also supported the hypothesis that the newly duplicated m6A regulatory genes have complementary features to the biological functions of their ancestral genes.

In general, gene duplication events, especially WGD accompanied by chromosomal rearrangements, are the major force acting upon the expansion of m6A regulatory genes and lay the foundation for triggering the functional innovation of these genes. Subsequently, the combined variations in gene sequence and expression abundance of m6A regulatory genes may further enrich the functional diversification of these duplicated gene members in tea plants. These factors coordinately facilitated the more explicit roles of m6A regulatory genes in different plant tissues and environmental adaptations and may contribute to the formation of a precise m6A regulatory mechanism in tea plants.

### The m6A Regulatory Genes Act as Essential Roles in Resistance Against the Environmental Stresses and Tea-Withering Stage

Accumulating evidence from prior reports uncovered that m6A modification displays high sensitivity and plays an essential role in response to various abiotic stresses (Hu et al., 2019; Zheng et al., 2020). In the present study, a large number of *cis*-acting elements related to stress responsiveness were presented in the promoter regions of major m6A regulatory genes, indicating that m6A regulatory genes may be involved in stress response. Two m6A writer genes, *CsMTB1* and *CsMTC*, exhibited sustained descending expression trends under drought stress, whereas the transcript levels of most m6A writer genes were marginally decreased (Figure 9). Likewise, only *CsMTA1* and *CsMTA2* were significantly downregulated during the late stage of cold stress. The expression levels of other m6A writer genes were not significantly modulated. On the contrary, the majority of m6A eraser genes were upregulated under drought and cold stresses, which were consistent with the expression patterns of these genes detected in *Arabidopsis* and rice under environmental stresses (Ma et al., 2006; Merret et al., 2015; Anderson et al., 2018). Combined with no significant alteration in most m6A writer genes, the upregulation of m6A eraser genes was considered to be responsible for the dynamic changes of RNA methylation level in tea plants under environmental stress. Global m6A hypomethylation was detected in maize under drought stress, which has been highlighted as a crucial contributor in the regulation of drought resistance (Miao et al., 2020). Therefore, we speculated that the dynamic changes of RNA methylation level in tea plants played indispensable roles in response to environmental stresses.

Reportedly, the m6A reader can recognize methylated transcripts and further regulate their fates through mRNA processing (Wang et al., 2015; Roundtree et al., 2017). In our research, different from m6A writer and erasers genes, a greater number of m6A reader genes exhibited significant expression variations in response to abiotic stresses. In particular, *CsECT8* and *CsCPSF30* belonging to the YTHDC subfamily were induced rapidly and persisted at high expression levels under drought and cold stresses. Previous studies proposed that YTHDC subfamily is mainly responsible for interacting with splicing factors to mediate AS events in the nucleus (Xu et al., 2014; Xiao et al., 2016). Moreover, AS is known to be one of the main contributors to posttranscriptional gene regulation *via* producing multiple mRNA transcripts and enhancing their coding potential from the same gene (Filichkin et al., 2015). In the plant kingdom, plants also trigger AS events to generate a large amount of stress-resistant mRNAs to cope with various environmental stresses (Cui and Xiong, 2015; Chen et al., 2020b). Notably, it has been recently observed that the increase in the number of AS events and differentially expressed AS transcripts contributes to the adaptation of tea plants to environmental stresses (Ding et al., 2020; Li et al., 2020b). Accordingly, we inferred that upregulation of *CsECT8* and *CsCPSF30* may be the upstream factors that activate the AS regulatory mechanism and further enhance the resistance of tea plants under abiotic stresses through regulating the transcript abundance of AS-induced isoforms.

Similar to the effects of abiotic stress on tea plants, FL were exposed to various abiotic stresses during the withering stage. Therefore, the underlying mechanism of m6A regulatory genes during the withering process also needs in-depth dissection. In this study, almost all of m6A eraser genes were insensitive after withering treatments, whereas the expression level of *MTC* was dramatically plummeted at IW and SW (Figure 10). In contrast, the transcript levels of six m6A eraser genes and three m6A reader genes were substantially elevated in IW and SW than in FL. Consistent with the environmental stress, withering also significantly altered the transcript levels of m6A regulatory genes. In IW vs. SW comparison, the up-regulation of m6A gene expression in SW may be due to the fact that solar-withering accelerated the dehydration of tea leaves, which is more severe than the stress experienced by leaves withered indoors. Reportedly, epigenetic mechanisms are widely implicated in abiotic stress response, including high temperature and UV radiation (Chang et al., 2020). Concomitantly, the significant alteration in global m6A level was detected after high-temperature and UV radiation treatments (Xiang et al., 2017; Scutenaire et al., 2018; Li et al., 2020c; Liu et al., 2020). Therefore, we speculated that m6A regulatory genes with a different expression between these two withering treatments may play the roles in response to the high-temperature and UV radiation. Additionally, it was noteworthy that *CsECT8* and *CsCPSF30* were also highly expressed in SW than in FL and IW, suggesting that the withering process may also produce a large number of AS events. As expected, we also observed that the number of AS events in SW was tremendously higher than that of FL and IW, clearly indicating that transcript abundance of these two genes was positively correlated with AS number. It has also been reported that AS is not only considered to be a vital role in response to various environmental stresses but also involved in the biosynthesis of secondary metabolites (Yuan et al., 2009; Xu et al., 2017; Liu et al., 2018). Flavonoids are characteristic metabolites associated with the bitterness and astringency of tea flavor, and their accumulation in tea plants has also been found to be regulated by AS events (Zhu et al., 2018b). Moreover, a previous study found that the flavonoid content in SW was significantly lower than that in FL and IW (Zhu et al., 2019). Accordingly, we speculated that m6A regulatory genes may be implicated in flavonoid metabolism through AS regulatory mechanism, thereby affecting the formation of tea flavor during the withering process. To validate this hypothesis, we performed the gene silencing on representative m6A regulatory genes (*CsMTC*, *CsALKBH9*, and *CsECT8*), and examined the flavonoid content in the related gene-silenced tea leaves, respectively. We observed that only the downregulation of *CsECT8* significantly suppressed the flavonoid accumulation after *CsECT8*-silenced treatment (Figure 12). These results hinted that, combined with the inhibition of flavonoid biosynthesis triggered by the withering process, high expression levels of m6A reader genes were considered to be mainly responsible for the suppression of flavonoid biosynthesis *via* modulating AS regulatory mechanism. Simultaneously, downregulation of m6A writer genes and upregulation of m6A eraser genes also synergistically reduced the flavonoid content. In addition, previous reports have shown that decreased flavonoid content may help form a weakly bitterness and mellow taste in tea infusions (Scharbert et al., 2004; Zeng et al., 2020). Consequently, m6A regulatory genes may also play crucial roles in improving the tea palatability during the withering process, which also confirms that solar-withering is superior to indoor-withering regarding the development of a premium flavor in oolong tea.

### The Complex Interplay Between RNA Methylation and DNA Methylation Contributes to the Flavor of Oolong Tea

In the central dogma, RNA is a bridge connecting DNA and protein to flow genetic information. Previous reports have shown that DNA methylation modifications occur on genomic DNA, and the modified DNA may be subject to RNA methylation for another round of reversible chemical modifications after being transcribed into RNA (Fu et al., 2014). Furthermore, our results showed that overall levels of 5mC and m6A contained a similar dynamic trend, and both of them have declined significantly after withering (Figure 11). It is noteworthy that the expression levels of several RNA MT genes were positively correlated with those of DNA MT genes. Likewise, the transcript levels of some RNA demethylase genes and DNA demethylase genes were highly correlated during tea-withering. Therefore, we speculated that there may exist a complex correlation between RNA methylation and DNA methylation during tea-withering process. Remarkably, the suppression of *CsMTC* significantly reduced m6A and 5mC levels, whereas both m6A and 5mC levels were markedly increased after *CsALKBH9*-silenced treatment. These results suggested that significant changes in the expression levels of m6A writer and eraser genes were not only involved in regulating the m6A level but also involved in modulating 5mC level.

**Figure 13:**
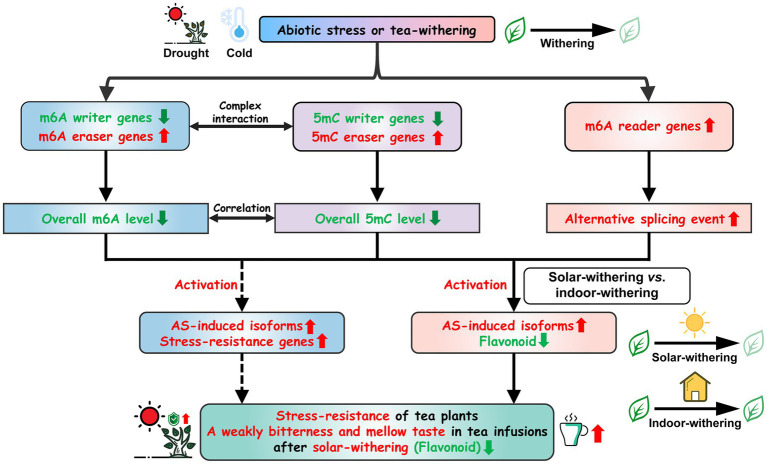
Schematic model for the roles of m6A regulatory genes in tea plants under abiotic stresses and tea-withering. Arrows represent the direct interaction; dotted arrows represent the indirect interaction.

As previously reported, the promoter regions of major *SlALKBHs* in tomato harbored distinct differentially DNA methylated regions, and hypermethylation of *SlALKBHs* substantially repressed their transcript levels (Zhou et al., 2019a). Meanwhile, accumulating evidence revealed that DNA methylation is highly interconnected with histone modifications at the posttranscriptional level (Sonmez et al., 2011; Jabre et al., 2019; Tian et al., 2019). More specifically, H3 methylation and DNA methylation in rice have a synergistic effect on suppressing gene expression (Zhou et al., 2016). Reportedly, RNA methylation is considered to be associated with chromatin remodeling *via* the combination of *ALKBH10* with multiple H3 modification markers (Jeong et al., 2009; Scarrow et al., 2020). These findings indicated that DNA methylation can be linked with RNA methylation through complex interactions. In addition, it is evident from a previous study that RNA demethylase genes can mediate the m6A modification status of DNA demethylase genes, thereby modulating its mRNA abundance, while DNA demethylase genes can influence the DNA methylation levels in the promoter regions of RNA demethylation genes (Zhou et al., 2019a). Data from this study also showed that transcript abundance of RNA demethylase was positively correlated with that of DNA demethylase genes during the withering process, whereas the expression levels of these demethylase genes were negatively correlated with the 5mC level and m6A level, respectively. Therefore, high expression levels of DNA demethylase genes in SW can stimulate the transcription of RNA demethylase genes by removing the DNA methylation marks, thereby indirectly inhibiting the flavonoid biosynthesis and improving the palatability of oolong tea. This helps to explain why solar-withering is beneficial to the production of high-quality oolong tea. Accordingly, we inferred that RNA methylation and DNA methylation formed a negative feedback by interacting with each other’s methylation regulatory genes, and the intertwined connection between DNA methylation and RNA methylation contributed to oolong tea flavor. In summary, a possible model is proposed to explain the biological functions of m6A regulatory genes in resistance against the environmental stresses and tea-withering stage (Figure 13).

## Conclusion

On the basis of the chromosome-scale genome of tea plants, we conducted a comprehensive genome-wide survey of m6A regulatory genes in tea plants for the first time. A total of 34 m6A regulatory genes were ultimately identified and grouped into three categories, namely, m6A writers, erasers, and readers. Then, we systematically analyzed chromosomal distribution, and gene duplication, and evolutionary selection of these m6A regulatory genes. In total, 30 m6A regulatory genes were randomly distributed on 13 chromosomes, and the remaining four genes were located on the unanchored contigs. We also found that segmental duplication and WGD events are the main contributors to the expansion of m6A regulatory genes. Additionally, gene structure analysis revealed instead of variation in conserved domain and motif, the gain or loss of exons and introns in gene sequence may contribute to the functional diversification of m6A regulatory genes. Subsequently, we detected the expression patterns of the identified m6A regulatory genes under environmental stresses and the tea-withering process. The results suggested that the m6A regulatory genes act as essential roles in resistance against the environmental stresses and tea-withering stage. Remarkably, we found that RNA methylation and DNA methylation formed a negative feedback by interacting with each other’s methylation regulatory genes, and high expression levels of DNA demethylase genes can stimulate the transcription of RNA demethylase genes by removing the DNA methylation marks, thereby indirectly inhibiting the flavonoid biosynthesis and improving the palatability of oolong tea. This study provided a solid framework for exploring the diverse functions of m6A regulatory genes in tea plants under environmental stresses and opened up new perspectives for understanding m6A-mediated regulatory mechanism on the improvement of tea palatability during the withering stage of postharvest processing.

## Data Availability Statement

The original contributions presented in the study are included in the article/Supplementary Material, further inquiries can be directed to the corresponding authors.

## Author Contributions

YG, ZL, and CZhu conceived and designed the study. YG, CZhu, and SZ performed the experiments and wrote the manuscript. YG, CZhu, SZ, CZhou, SX, GC, CT, and KX analyzed the experimental data. CZhu, SZ, CZhou, and YL performed the plant treatments and revised the manuscript. All authors have read and approved the manuscript.

### Conflict of Interest

The authors declare that the research was conducted in the absence of any commercial or financial relationships that could be construed as a potential conflict of interest.

## Supplementary Material

Supplementary Figure S1 Sequence logos of conserved motifs in m6A regulatory genes **(A)** m6A writer genes; **(B)** m6A eraser genes; **(C)** m6A reader genes.
