# Supplementary figures and images for: Genome-Wide Investigation of N6-Methyladenosine Regulatory Genes and Their Roles in Tea (Camellia sinensis) Leaves During Withering Process
Source: Front Plant Sci. 2021 Jun 15;12:702303. doi: 10.3389/fpls.2021.702303 (PMC8240813; doi:10.3389/fpls.2021.702303)

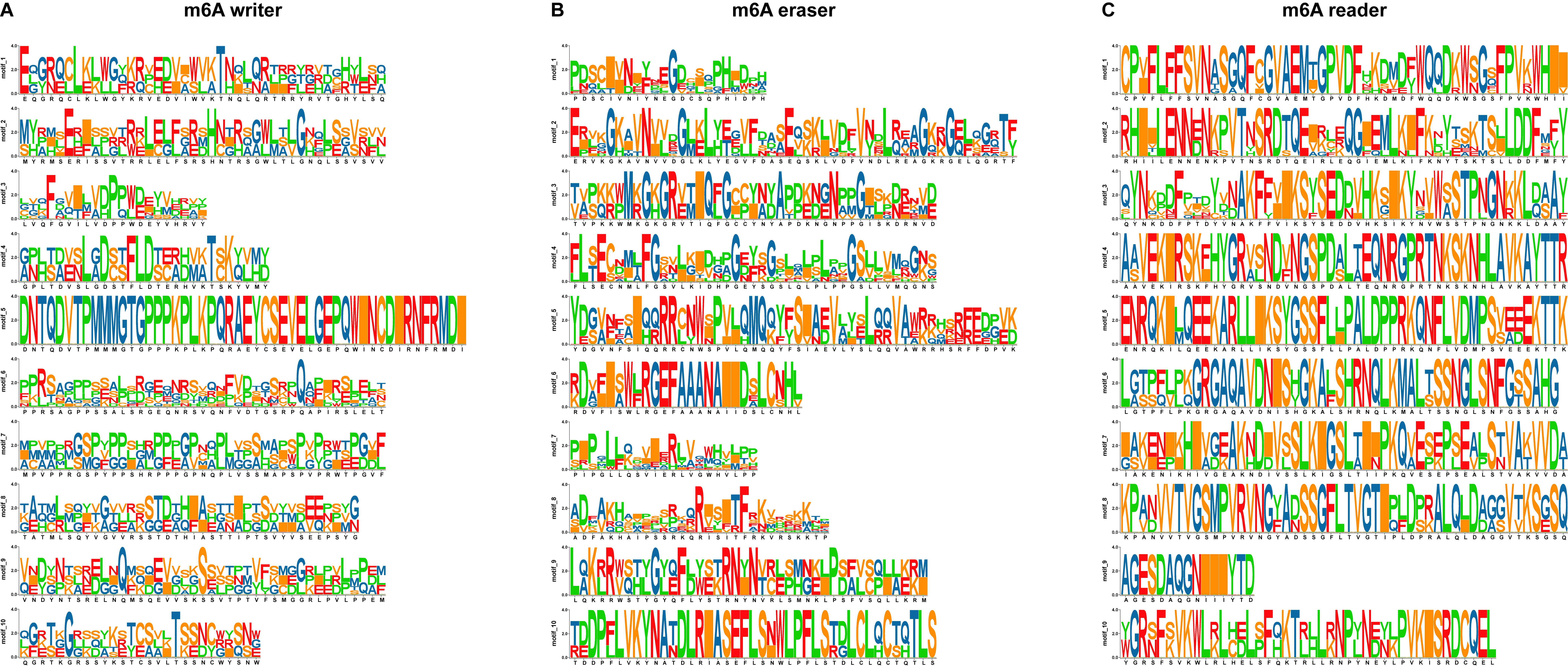

Supplement: Supplementary Figure S1 — Sequence logos of conserved motifs in m6A regulatory genes (A) m6A writer genes; (B) m6A eraser genes; (C) m6A reader genes. [file Image_1.TIF]
